# Retrospective

**Published:** 1861-01

**Authors:** 


**RETROSPECTIVE.**—It is not our purpose to dye ourselves or our readers with indigo this opening month of another year. Blue reminiscences are, for the most part, unremunerative. A hearty, whole-souled, wide-mouthed laugh is incomparably more healthful; it enlivens the circulation, mollifies the heart, and wakes us up to newness of life. The retrospect we have in view is the turning back to the days when our JOURNAL was young, and glancing at some of its performances. Among other things, it introduced to its patrons, then a little band, the most wonderful medicines—"Patent" of course —and described "to our hand" by some abler pen. Wonder if it now "lies silent in the grave?" If so, here's a thought to thy memory, genial heart, with a hope that a purer and heavenlier gladness plays upon your face "on the other side."

